# A Novel Automatic Rapid Diagnostic Test Reader Platform

**DOI:** 10.1155/2016/7498217

**Published:** 2016-04-14

**Authors:** Haydar Ozkan, Osman Semih Kayhan

**Affiliations:** ^1^Electrical Engineering Department, University of California, Los Angeles, CA 90095, USA; ^2^Biomedical Engineering Department, Fatih Sultan Mehmet Vakıf University, 34445 Istanbul, Turkey; ^3^Biomedical Engineering Department, Istanbul Technical University, 34467 Istanbul, Turkey

## Abstract

A novel automatic Rapid Diagnostic Test (RDT) reader platform is designed to analyze and diagnose target disease by using existing consumer cameras of a laptop-computer or a tablet. The RDT reader is useable with numerous lateral immunochromatographic assays and similar biomedical tests. The system has two different components, which are 3D-printed, low-cost, tiny, and compact stand and a decision program named RDT-AutoReader 2.0. The program takes the image of RDT, crops the region of interest (ROI), and extracts the features from the control end test lines to classify the results as invalid, positive, or negative. All related patient's personal information, image of ROI, and the e-report are digitally saved and transferred to the related clinician. Condition of the patient and the progress of the disease can be monitored by using the saved data. The reader platform has been tested by taking image from used cassette RDTs of rotavirus (RtV)/adenovirus (AdV) and lateral flow strip RDTs of* Helicobacter pylori* (*H. pylori*) before discarding them. The created RDT reader can also supply real-time statistics of various illnesses by using databases and Internet. This can help to inhibit propagation of contagious diseases and to increase readiness against epidemic diseases worldwide.

## 1. Introduction

Investigation and detection of symptomatic and asymptomatic diseases have been improved via Rapid Diagnostic Tests (RDTs) or lateral immunochromatographic assays, since the beginning of last century. RDT is quick, easy to perform, and used for early, fast, and precise detection of some diseases to prevent undesired harms of diseases, to aid in epidemic readiness, and to inhibit long-term complications of diseases for public health risks [1–5]. The certain public health problems comprise more effective monitoring of chronic conditions, contagious disease, and drug of abuse, in risky areas, and they can be administrated by low-experienced trained medical personnel. Chromatographic immunoassays are counted as the most-used rapid tests or screening tests, which indicate the presence or absence of an analyte in an organic material. The result is available within a matter of minutes without involving the use of special processes or instruments. Various diseases such as AdV, RtV,* H. pylori*, hepatitis, HIV, and malaria and certain physiological conditions such as cholesterol level, blood sugar, drugs of abuse, and pregnancy can be diagnosed via RDTs [6]. Moreover, the RDTs have become essential tools to diagnose some illnesses when facilities and expertise such as standard laboratory tests, clinical examination, microscopy, medical practitioner, and technician are inadequate [7–11].

Whilst RDT technology has been developing, the utilization of personal computer and tablet technology has gradually enhanced in terms of medical purposes. The quality of healthcare can be advanced to minimize and avoid mistakes in clinical work by using computer based decision support (CBDS) for diseases [12, 13]. The evaluation of RDT is usually analyzed by direct visual inspection, instead of CBDS. Thus, the interpretation of results can differ depending on the clinical technician's skills, training, and experience, also lighting conditions of environment, and so forth. Therefore, analysis of RDT has some restrictions. One of the limitations of analyzing RDTs manually is that tentative test line can sometimes be overlooked and evaluated as false negative. Another one is that the decided results are stored usually in a paper-based format or typed to a computer manually. Especially in a large-scale hospital, manual result reporting is time consuming. Time dissipation can be a problem aspect of accurate reading since visibility of test line on the RDT disappeared gradually.

Recent years have seen an extensive research and development effort to realize automatic digital test readers which can carry out qualitative [14] as well as quantitative [15, 16] analysis. The RDTs such as lateral flow strips [17, 18], cassette test [19], pads [20], dipstick [21], and microfluidic chip immunoassay [22] have been analyzed with digital test readers. However, they usually work with specific set of RDTs or limited number of RDTs from a particular manufacturer. Unfortunately, they are still outsize and expensive. There are recent developments on smartphone based RDT readers to work with different types of RDTs [6, 23–26]. In addition, Google Glass based RDT reader has been introduced as an advantage of wearable technology [27]. However, some of mobile RDT readers are still expensive technology since they use external hardware such as battery, light emitting diode (LED), lens, and microcontroller on the 3D attachment and Google Glass is expensive device indeed. Besides, during the RDT reading via cell phone and Google Glass, motion artifact image can occur. The cell phones are held and at the Google Glass based reading platform, the RDT is grabbed and Google Glass is worn on human eyes. The hands and human heads can shake during the image capturing.

In this study, we designed laptop-computer and tablet based RDT automatic reading system that can be easily integrated on an existing hospital system (laptop, tablet, database, server, etc.) and tested RtV/AdV and* H. pylori* RDTs. Medical personnel can access these systems more effectively and learn how to use them in a short period of time due to user-friendly interfaces of the RDT-AutoReader 2.0. The automatic reading platform is feasible and low-cost since the system has small, cost-effective, and compact mechanical 3D attachments without any external battery, lens, or illumination source. The cassette RDT holder with RtV/AdV RDT is placed in either front or back of the existing camera of a computer or tablet. The lateral flow strip RDT is firstly inserted into the strip holder and the strip holder is then inserted into the cassette RDT holder. Using the designed decision program, the image of RDT is taken, the area which covers control line and test line is cropped as ROI, and the average intensity spectrum is drawn to define the validation and decision. At the same time, features from test line are extracted for classification of presence of disease using support vector machine (SVM). The decision is classified through both average intensity spectrum and SVM having high accuracy as 100%. The designed reader platform is inexpensive and there is no image motion artifact since both computer and RDTs are not grabbed. Test repeatability, accuracy, and standardization are provided owing to the attachments that can be convenient for majority of laptop-computers and tablets. The developed RDT-AutoReader can work with various lateral immunochromatographic assays and yield their quantitative results as invalid, positive, or negative. Furthermore, the RDT images and the patient reports are saved on the memory of computers; entire data is stored in the hospital database and in a cloud server and also transferred to the clinicians. All the process works in real-time and medical technicians will be saving time and more patients can be diagnosed in a day via fast and electronic recording system once the customized RDT-AutoReader is used.

## 2. Materials and Methods

### 2.1. Dataset

**Figure 1:**
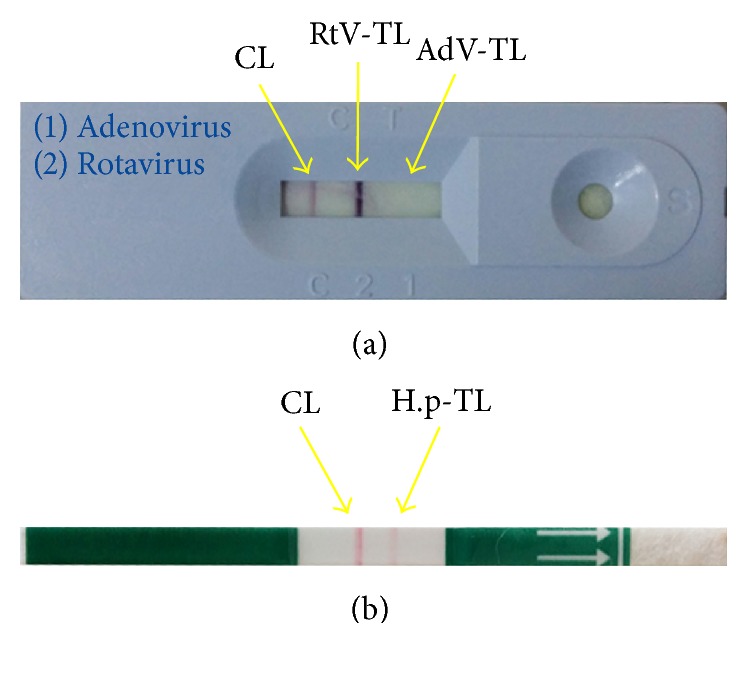
(a) The cassette-based RDT having positive RtV result. (b) The lateral flow strip RDT having positive* H. pylori* result.

In this study, experimental RDT images have been taken from Istanbul University Hospital. Routinely, RtV/AdV cassette-based RDTs and* H. pylori* lateral flow strip RDTs are done in the hospital and the used RDTs are discarded after visual inspection manually by the medical technicians.  Figure 1(a) shows a processed cassette-based RDT of RtV/AdV. The lines on the left side, the middle, and the right side of the ROI are control line (CL), rotavirus test line (RtV-TL), and adenovirus test line (AdV-TL), respectively. A processed lateral flow strip RDT of* H. pylori* is shown in Figure 1(b). The lines on the left side and the right side of the ROI are CL and* Helicobacter pylori* test line (H.p-TL), respectively. The cassette-based RDTs have been inspected as positive RtV with visual inspection by medical technicians undoubtedly since the RtV-TL is seen clearly (Figure 1(a)). However, the lateral flow strip RDT is evaluated as negative by some of the technicians since the H.p-TL is seen tentative (Figure 1(b)). Our automatic decision system has read both RDTs as positive. After digital reading, the medical technicians have inspected the lateral flow strip RDT again carefully and they have corrected their previous visual evaluation result and decided that the result is positive.

The image has been captured from used RDTs and analyzed digitally by comparing with visual evaluation. RtV are prevalent and lead to acute diarrheal illnesses for adults, children, and newborns [28]. Human AdV, one of the main reasons of different illnesses, are widespread around the world. Diseases such as encephalitis, pneumonia, diarrhea, hepatitis, myocarditis, conjunctivitis, and cystitis caused AdV contaminations in routine medical symptoms [29].* Helicobacter pylori* is related to diseases such as gastritis, ulcer, lymphoid, gastric and adenocarcinoma and other gastroduodenal diseases [30].

### 2.2. The Automatic RDT Reader Platform

**Figure 2:**
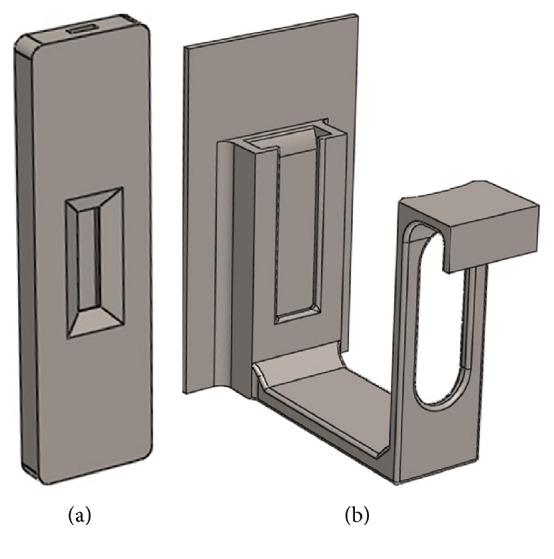
3D-printed attachments: (a) lateral flow strip holder whose shape is as an existing cassette RDT and (b) the cassette RDT holder. In the strip holder, there is a channel to put strip RDT and the ROI area is just seen after inserting the strip RDT. The lateral flow strip is inserted into the strip holder from the top channel toward bottom and the strip holder is inserted into the cassette RDT holder to analyze* H. pylori*. The RDT of RtV/AdV directly inserted into the cassette RDT holder.

**Figure 3:**
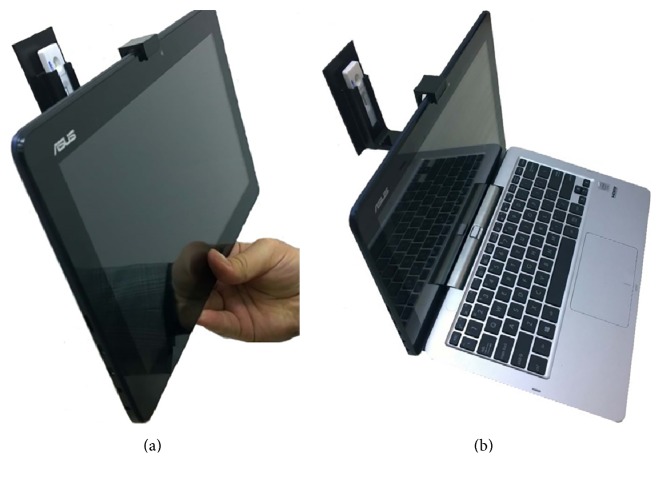
(a) A view of a tablet and worn RDT holder. (b) A view of a laptop and worn RDT holder.

**Figure 4:**
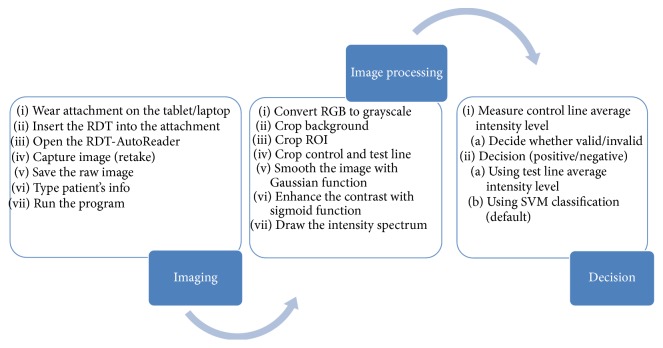
The steps of the created RDT reader platform processes.

An RDT reader platform which is aimed at running on laptop-computers, tablets, or 2-in-1 computers has been designed. The RDT reader platform consists of a lateral flow strip RDT holder (Figure 2(a)), a cassette RDT holder (Figure 2(b)), and also a newly designed customized program named RDT-AutoReader 2.0. The holders are 3D-printed, low-cost, tiny, and compact devices and are adaptable to most tablets or laptop-computers. The cassette RDT holder can be worn on the camera of a tablet or a laptop (Figure 3). The main aim of the holder is to hold and fix the RDTs so that camera can be focused clearly. Neither the computer nor the RDTs are held with hand. This feature makes it possible to take clear images without motion artifacts. The system analyzes the RtV/AdV RDTs by using the cassette-based RDT holder and the* H. pylori* RDTs by using both the strip holder and the cassette RDT holder. The detailed steps of the designed RDT reader platform processes are shown in Figure 4 with the sections of imaging, image processing, and decision.

**Figure 5:**
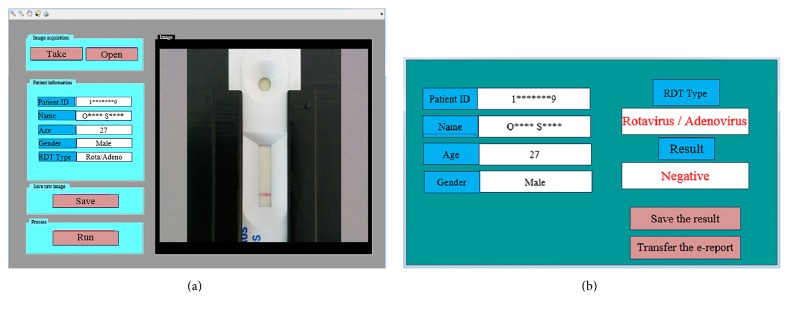
A view of RDT-AutoReader program: (a) process interface and (b) result showing interface.

First of all, RDT holder is placed to face either front or back camera of a computer or a tablet. A cassette RDT or the designed strip holder with a strip RDT is inserted into the RDT holder and RDT-AutoReader application is run. To avoid contamination of the holder, it is best to keep the sample well at the upper side. Namely, control line of the RDT should be placed at the lower side. The RDT-AutoReader program is designed on MATLAB graphical user interface (GUI) and transformed to a user-friendly program through MATLAB compiler. The RDT-AutoReader has two interfaces which are run on the processes (Figure 5(a)) and display the result with patient information (Figure 5(b)). The user takes the image of the RDT by pushing the “Take Image” button on the interface. The raw image can be retaken and saved. Meanwhile, the related data about patient such as patient ID, name, age, gender, and RDT type are filled in the gaps on the interface. The user pushes the “Run” button to operate all image processing, machine learning, and electronic report system automatically. No Internet connection or extra device is required for processing phase of the system.

### 2.3. Imaging and Image Processing

The computer and tablet cameras can take images in different formats. One of the formats is Red-Green-Blue (RGB) that consists of 3 channels and 24 bits (8 bits per channel). Another color space is YUV format (YCbCr; Y: value of luminance, Cb: Chrominance blue, and Cr: Chrominance red) which is also 3 channels and 24 bits indeed. First the RGB or YUV images have been converted to 8-bit grayscale images since a gray level image is adequate to differentiate contrast differences of control and test lines. This process offers to reduce complexity and cost of operations. Subsequently, the image has been separated as holder part and background part by cropping. After discarding background part, the corners of holder have been found using Harris corner detection method and cropped the ROI using the locations of the corners directly. Furthermore, a rectangle has been determined around the control and test lines and cropped them.

According to pertinent technical sources, one of the prominent difficulties is overlooking tentative test line. To overcome this problem, some spatial filtering algorithms have been implemented to make test line more visible. All ROIs have random background noise. The noises have been removed from the ROI by applying averaging filter on image. 2D Gaussian averaging filter has been implemented to reduce the noise, normalize the intensity levels, and fix diluted areas. The probability distribution function of Gaussian random variable *x* is given by

(1) $$\begin{array}{c} \rho \left(\;x\;\right)=\frac{1}{\sigma \sqrt{2\pi }}e^{-{\left(x-\mu \right)}^{2}/2\sigma^{2}}, \end{array}$$

where *x* symbolizes intensity value, *μ* is average (mean) value of *x*, and *σ* represents standard deviation of *x*. Square of standard deviation (*σ* ^2^) is called variance. To estimate the mean and variance value of pixels on image,

(2) μ=1MN∑x=0M−1 ∑y=0N−1fx,y,σ2=1MN∑x=0M−1 ∑y=0N−1fx,y−μ2.

Equations (2) have been used. Gaussian filters are usually isotropic (circularly symmetric), whose standard deviation is the same along both dimensions. The filter has been adjusted by changing the standard deviation value. After implementation of Gaussian averaging filter, intensity manipulation is needed to increase the visibility of the control and test lines. Sigmoid function is utilized to enhance contrast of desired pixel range as follows:

(3) $$\begin{array}{c} f\left(\;x,a_{1},a_{2},a_{3}\;\right)=\frac{a_{1}}{1+e^{\left(-a_{2}x\right)}}-a_{3}, \end{array}$$

where *a*_1_, *a*_2_, and *a*_3_ are dynamic range, slope, and bias of the function, respectively. As a result, we could reveal the lines tractably, especially for fade test lines, to improve accuracy of test outcomes.

**Figure 6:**
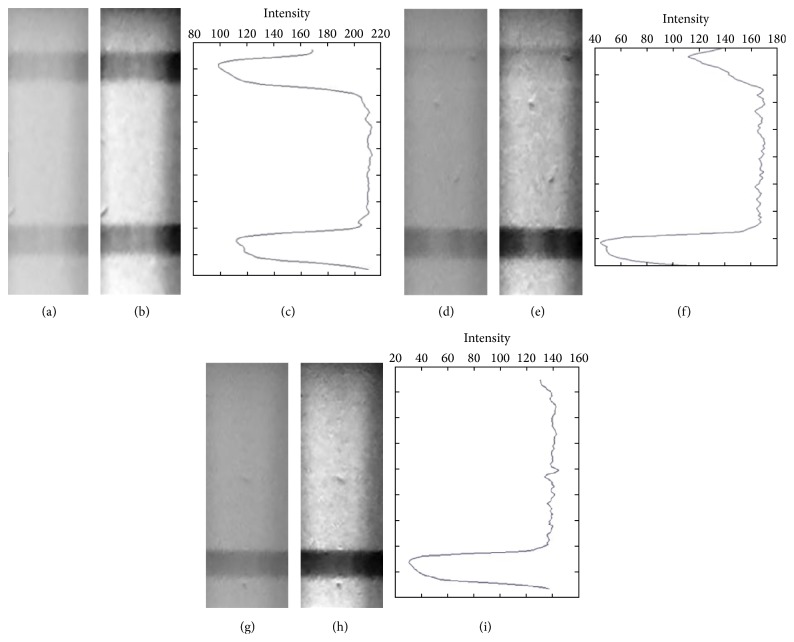
Processed ROI images. (a) Positive raw captured image. (b) Contrast enhanced ROI of (a). (c) Average intensity spectrum of ROI of (b). (d) Tentative positive raw image. (e) Contrast enhanced ROI of (d). (f) Average intensity spectrum of ROI of (e). (g) Negative raw captured image. (h) Contrast enhanced ROI of (g). (i) Average intensity spectrum of ROI of (h).

During the last stage of image processing operation, an intensity spectrum has been drawn according to each row of ROI line. This process can be called one-dimensional line averaging filter. First of all, entire column intensity values per row of ROI are averaged and placed in a vector with sequence of row line, respectively. This vector consists of whole characteristic of image. Finally the average intensity spectrum is plotted. Mean intensity spectrum gives the lowest peak intensity values according to number of test lines. The original images of ROIs that have positive, tentative, and negative test lines are seen, respectively, in Figures 6(a), 6(d), and 6(g). After Gaussian and sigmoid transformation techniques, Figures 6(b), 6(e), and 6(h) are achieved. After contrast enhancement, it is clearly seen that the test lines visibility is increased. This benefit especially lets the user not to overlook the tentative test line. Also the intensity spectrums using 1D line averaging method are obtained and shown in Figures 6(c), 6(f), and 6(i). The spectrums have significant distinctive features to classify the presence of diseases.

### 2.4. The Decision Processes

The decision process yields three results, namely, invalid, positive, and negative. To eliminate the RDT being invalid, the intensity peak level of control line has been measured after drawing the average intensity spectrum of ROI. The intensity level must be lower than predetermined threshold level (*T*_1_). If it is above *T*_1_, the system warns the user displaying the message of “RDT is invalid.” If the RDT is valid, two different methods are used for deciding the test result. First, the average intensity peak value of the test line has been measured and if the value is lower than the defined threshold level (*T*_2_), the system marks that the result of RDT is positive; otherwise, the result is negative. Second, the automatic reader system has extracted features such as average, maximum, minimum, standard deviation, and max-min difference from cropped test lines. Using the 50% of mentioned features for training and 50% for test data, the result has been classified by radial basis SVM. The decision and patient's information have been saved in the computer and the e-report has been generated and transferred to the clinician network folder and cloud.

### 2.5. Electronic Recording and Transferring Processes of the RDT Results

After diagnosis of test, the test result is recorded in the computer, in the local network, and in the cloud. First of all, the medical technician should click the “Save the Result” button on the interface to save patient's test result with related patient's information into an excel sheet in the laptop or tablet. The saved data in a row of the excel sheet includes the following: (1) previously typed patient's protocol ID, name, age, gender, and RDT type; (2) the date and time which are read from computer automatically; (3) the analyzed RDT result electronically. The mentioned patient's information with specific date, time, and result is automatically stored from RDT-AutoReader to the excel column, respectively. When the next test reading is completed digitally, all information of new patient is replaced in the next row of the excel sheet sequentially.  Table 1 shows an electronic recording sheet with five patients' information and their results. In the light of keeping the whole information, public health records can be analyzed in terms of the disease, gender, and age. Additionally, storing the specific date and time automatically will generate statistical database to track the health status such as propagation of related diseases in specific seasons, months, or years. When the technician clicks the “Transfer the e-report” button, patient data is sent to the folder of patient's clinician. Two protocols are used to transfer the data. (1) The data is transferred to the clinician folder in the local network. (2) The data is sent to the created cloud network as real-time. Thus, the clinician could check the result without time-free and location-free. As a consequence, clinicians can track their patients and generally propagations of the diseases observing the generated database.

## 3. Results and Discussions

The performance of designed RDT reader platform has been validated by testing the lateral flow-based RDTs and the cassette-based RDTs which detect* H. pylori* and RtV/AdV, respectively. The designed RDT reader system captures and processes the RDT images and then classifies the test results as invalid, positive, or negative. First of all, the total of 281 RDT tests has been evaluated and reported. 119 of 281 are positive, 137 of 281 are negative, and the rest (25) are invalid (Table 2). The test results have been verified through visual inspection by medical technicians. The automatic evaluation system contains two methods which are average intensity spectrum analysis and SVM classification. Both methods provide high accuracy as 100% and the SVM method is assigned as default. Additionally, Table 2 includes statistical information where the number of positive* H. pylori* is higher than both the numbers of positive AdV and RtV. Through this significant information, the progress of public health status can be controlled.

In the literature, there are several cell phone based and Google Glass based RDT reader platforms as point-of-care (POC) applications. They have proposed different methods to classify RDTs as positive, negative, or invalid automatically. Carrio et al. have used a multilayer perceptron artificial neural network classification technique to classify the RDT and achieved 96% accuracy [6]. Mudanyali et al. have measured color intensity level of test and control line to read RDT automatically and demonstrated 100% accuracy [23]. Shen et al. have used colorimetric diagnostic assays and quantified the colors. They declared that their study has high accuracy without specific number [24]. You et al. have evaluated their reading system by using intensity level of test end control line having 97% accuracy [25]. Dell and Borriello's study needs a health worker who has technical experience to identify and save a template image for each type of RDTs. The working system of mechanism requires alignment between template image and image taken by device before the process. They have used threshold based decision system and reported approximately 97% accuracy [26]. Feng et al. have used QR code to crop ROI and SVM to classify the RDT by having 100% accuracy [27].

Additionally, we have focused on the POC applications in terms of their significant advantages and disadvantages and compared them with our developed method (Table 3). First of all, most of the POC applications and our study have 3D attachments that can affect test repeatability, test accuracy, time consumption, illumination, and contamination [6, 23, 25, 26]. Since the attachment has a specific place for RDT, tests can have same standard, same possible desired image, and repeatability. However, if there is no attachment or the attachment is not stable, then motion artifacts can occur on image and this declines the image quality [23–27]. Carrio et al. [6] study and our study have common benefit of not having motion artifact since they have both stable image capturing platforms and RDT holders. Carrio et al., Mudanyali et al., and You et al. have external illumination platform which have some LEDs and batteries and this can provide environmental independency [6, 23, 25]. Although external illumination is a beneficial aspect of image quality, it is also costly. Our study does not have an external illumination source but the stable computer based reading platform is adjustable according to indoor lighting facilities. Having an external hardware on the 3D attachment has another disadvantage coupled with high-cost. When the sampled test is put to the RDT holder, the holder can be contaminated. At this situation, it is easy to disinfect the attachment if there are no additional electronic and optical systems on it.

As a virtue of the system, there are further important specifications such as processing on device, obtaining real-time data, and storing on the memory of device. Yielding result in a short period of time is essential especially for epidemic diseases. Therefore, processing on the device is substantial and necessary. Furthermore, if process could not run on the device and an external processing source is required as a server, the results would be delayed according to connection quality, server, and device performances. The results should be obtained in real-time. Although Google Glass RDT reader platform is a novel wearable technology, it needs a server for image processing and SVM techniques [27]. When there is no Internet or Bluetooth access to a server, the results will wait until the connection is provided. These situations are undesirable especially for emergency circumstances. Some readers can have not only qualitative tests but also quantitative tests such as pH tests [24], thyroid stimulating hormone tests [25], and prostate-specific antigen tests [27].

Other benefits of POC RDT reader applications are that they are protectable and can have easy information sharing technologies due to the improvement of network and Internet devices. One of the key advantages is provision of automatic progress and electronic report generation for patients. The e-reports can be saved and later sent to the clinician in charge. As a result, time consumption is reduced, productivity of medical technician is raised, and diagnosis and treatment efficiency is increased. Another merit of the reader platforms is that all the patient data such as name, RDT image, age, and gender and also system data such as place and time can be stored in a server or cloud system. There are three side benefits to keep all the data saved. Firstly, all patient information can be interpreted and tracing epidemic disease propagation becomes possible. Secondly, individual patient progress can be displayed and tracked over a period of time. Thirdly, the data can be used to educate students or unexperienced medical workers.

As a summary, we supplied test repeatability and high accuracy avoiding motion artifacts since the attachment and device are stable. The proposed reader platform does not have any illumination source but the system may work efficiently under indoor lighting conditions. Since our component does not have any electronic or optical features, it is inexpensive and the attachment can be disinfected easily and the used laptop or tablet will not be contaminated. Another advantage is that our system runs on laptop-computer or tablet device at all stages of processing in real-time without needing connection to a network or Internet. It provides an easy and quick decision mechanism. For automatic decision, the platform does not need expert medical workers. To parallel the other POC readers, our platform also produces e-reports and stores and sends them to the clinician network folder, server, and cloud. Additionally, our computer based automatic reading system can be easily inserted into an existing hospital computer system and it is the fastest system among those of the literature since the processors of computers are faster than the processors of smartphones and Google Glass.

## 4. Conclusion

A novel laptop-computer or tablet based RDT reader platform which is also a POC application has been demonstrated. The designed system comprises two attachments that are tiny, simple, low-cost, and 3D-printed and a customized reader program called RDT-AutoReader. The RDT-AutoReader captures the RDT images and processes and classifies them by removing the risks of visual reading. The captured raw RDT images and the decision result with patient's information are saved to the computer memory, stored in the hospital database and in the cloud server, and also transferred to the clinician network folder. The success of our reading platform has been demonstrated by taking image from processed* H. pylori* RDT and RtV/AdV RDT. After image processing and classification, we achieved excellent accuracy as 100%. Since all the process runs in real-time, medical technicians will be saving time and diagnose more patients in a day through this fast and digital recording RDT-AutoReader system. Moreover, storing the results of the disease electronically provides statistical information. This situation will contribute to control public health status. As a result, it has been observed that the performance of the designed RDT reader platform and its significant advantages in this study are better than the ones in the literature and the proposed RDT-AutoReader system could be recommended as highly practical to use as a digital POC reader or as a clinical reader in a hospital by the medical technicians.

## Figures and Tables

**Table 1:** The electronic recording of patient's information, specific date, time, and result of RDT.

Patient ID	Name	Age	Gender	Date	Time	RDT type	Results
1^*∗∗∗∗∗∗∗*^1	O^*∗∗∗∗*^ S^*∗∗∗∗*^	27	Male	Jan. 15, 2016	13:21:33	Adenovirus	Positive
1^*∗∗∗∗∗∗∗*^2	A^*∗∗∗∗∗*^ O^*∗∗∗∗*^	35	Male	Jan. 15, 2016	15:32:54	Rotavirus	Negative
1^*∗∗∗∗∗∗∗*^3	K^*∗∗∗∗*^ Y^*∗∗∗∗∗∗*^	25	Female	Jan. 16, 2016	14:18:22	*H. pylori*	Negative
1^*∗∗∗∗∗∗∗*^4	M^*∗∗∗*^ E^*∗∗∗*^ A^*∗∗∗*^	30	Male	Jan. 16, 2016	15:23:04	*H. pylori*	Positive
1^*∗∗∗∗∗∗∗*^5	D^*∗∗∗∗*^ M^*∗∗∗∗∗*^	28	Female	Jan. 17, 2016	16:14:43	*H. pylori*	Negative

**Table 2:** The number of evaluated RtV/AdV and *H. pylori* (H.p) RDTs and the accuracy of classification.

Evaluations											
	Tentative positive			Positive			Negative		Invalid		Accuracy
	RtV	AdV	H.p	RtV	AdV	H.p	RtV/AdV	H.p	RtV/AdV	H.p	
Correct	19	17	27	18	14	24	72	65	15	10	100%
Incorrect	0	0	0	0	0	0	0	0	0	0	0
Total	119 positive						137 negative		25 invalid		

**Table 3:** The significant advantages (Ad) and disadvantages (DA) of previous studies and our study.

Remark	References													
	Developed method		Mudanyali et al. [23]		Feng et al. [27]		Carrio et al. [6]		Dell and Borriello [26]		Shen et al. [24]		You et al. [25]	
	Ad	DA	Ad	DA	Ad	DA	Ad	DA	Ad	DA	Ad	DA	Ad	DA
(i) Point-of-care application	√		√		√		√		√		√		√	

(ii) Having 3D attachment	√		√			√	√		√			√	√	

(iii) Motion artifact image	√			√		√	√			√		√		√

(iv) Has an illumination source		√	√			√	√			√		√	√	

(v) Cost	√			√		√		√	√		√			√

(vi) Real-time processing on device	√		√			√	√		√		√		√	

(vii) Expertise requirement	√		√		√		√			√	√		√	

(viii) Quantitative tests		√		√	√			√		√	√		√	

(ix) Storing data and transferring e-report	√		√		√		√		√		√		√	

(x) Easy to integrate a hospital computer	√			√		√		√		√		√		√

(xi) Fast analysis	√			√		√		√		√		√		√
